# Adrenal function in rheumatoid arthritis: a correlation with disease activity

**DOI:** 10.1186/ar3628

**Published:** 2012-02-09

**Authors:** Richard Imrich, Miroslav Vlcek, Jana Kerlik, Michael Vogeser, Fabian Kirchhoff, Adela Penesova, Zofia Radikova, Jozef Lukac, Jozef Rovensky

**Affiliations:** 1 Laboratory of Human Endocrinology, Institute of Experimental Endocrinology, Slovak Academy of Sciences, Bratislava, 833 06, Slovakia; 2 Center for Molecular Medicine, Slovak Academy of Sciences, Bratislava, 831 01, Slovakia; 3 Institute of Clinical Chemistry, Hospital of the University of Munich, Munich, 81377, Germany; 4 National Institute of Rheumatic Diseases, Piestany, 921 12, Slovakia

## Background

Hypothalamic-pituitary-adrenocortical dysfunction contributes to a complex pathogenesis of rheumatoid arthritis (RA). Decreased production of adrenal androgens and subtle changes in cortisol production has been observed in RA, particularly in female patients with premenopausal onset of the disease. Our study was aimed to investigate (1) adrenocortical function in relation to disease and inflammatory activity and to analyze cortisol bioavailability in RA females.

## Materials and methods

Adrenal steroids including free plasma cortisol responses to the low-dose ACTH stimulation test (1 µg Synactheni.v.) were investigated in 23 premenopausal RA and in 15 age- and BMI-matched healthy females. Twelve (N = 12) out of 23 RA patients were on low-dose glucocorticoids (<8.5 mg/day of prednisone or equivalent). When patients were divided into low (disease activity score 28; DAS28≤ 3.2) and moderate to high disease activity (DAS28>3.2) subgroups, glucocorticoid-treated patients comprised 53% and 50% of patients in each of the subgroups. Plasma C-reactive protein, interleukin (IL)-1β, IL-4, IL-6, IL-8, IL-10, IL-17, interferon gamma and tumor necrosis factor alpha concentrations were measured at the baseline.

## Results

RA patients had high C-reactive protein, IL-6, IL-8 and tumor necrosis factor alpha. Patients with DAS28>3.2 had lower (p < 0.05) total plasma cortisol, 17-hydroxyprogesterone, dehydroepiandrosterone and androstenedione responses in the ACTH test compared to healthy controls. Patients with DAS28>3.2 had lower (p < 0.05) dehydroepiandrosterone response in the ACTH test compared to patients with DAS28≤ 3.2. C-reactive protein (CRP), DAS28, and interleukin (IL)-6 negatively correlated with androstenedione response to Synacthen. Responses of all measured adrenal steroids were lower (p < 0.05) in patients on low-dose glucocorticoids compared to healthy controls. RA patients not treated with glucocorticoids had lower total cortisol response (p = 0.038) compared to controls, however, these patients did not differ in free plasma cortisol in the ACTH test.

## Conclusions

The present data indicate an association of increased disease activity with a decrease in adrenal androgen-producing *zonareticularis*in RA. A modest suppression of stimulated cortisol in glucocorticoid-untreated RA patients is not associated with decreased cortisol bioavailability.

